# Inhibition of Hypoxia-Inducible Factor-1α and Vascular Endothelial Growth Factor by Chrysin in a Rat Model of Choroidal Neovascularization

**DOI:** 10.3390/ijms21082842

**Published:** 2020-04-18

**Authors:** Ji Hun Song, Ka Young Moon, Sung Chul Lee, Sung Soo Kim

**Affiliations:** 1 Department of Ophthalmology, Ajou University School of Medicine, 164 World Cup-ro, Yeongtong-gu, Suwon 16499, Korea; 2 Department of Ophthalmology, The Institute of Vision Research, Yonsei University College of Medicine, 134 Shinchon-dong, Seodaemun-gu, Seoul 03722, Korea

**Keywords:** age-related macular degeneration, chrysin, choroidal neovascularization, hypoxia-inducible factor-1 alpha, vascular endothelial growth factor

## Abstract

Age-related macular degeneration (AMD) is a leading cause of irreversible vision loss among the elderly population. Vascular endothelial growth factor (VEGF) is essential for choroidal neovascularization (CNV) development in advanced, wet AMD. Chrysin (5,7-dihydroxyflavone) is a natural flavonoid with anti-inflammatory, anti-oxidative, and anti-angiogenic effects. We hypothesized that intravitreally injected chrysin may inhibit CNV due to its inhibitory effect on angiogenesis. To determine the effects of chrysin on an experimental CNV model, we induced CNV in Brown Norway rats with a diode laser. One week later, rats were injected intravitreally with chrysin in the right eye and vehicle in the left eye. The following week, we evaluated chrysin’s effects via the CNV grade assessed with fluorescein angiography and histologic analyses. Hypoxia-inducible factor-1 alpha (HIF-1α) and VEGF expression in the retina/choroid complex were also measured in both eyes. The mean CNV grade was significantly lower in chrysin-treated vs. control eyes (2.34 ± 1.14 vs. 2.97 ± 1.05, *p* < 0.001), as was the mean CNV thickness (33.90 ± 4.89 vs. 38.50 ± 5.43 μm, *p* < 0.001) and mean HIF-1α and VEGF levels (both *p* < 0.001). Compared to chrysin-treated eyes, the relative risk of control eyes developing high-leakage lesions was 2.03 (95% confidence interval: 1.46–2.83). Since chrysin inhibited laser-induced CNV and downregulated HIF-1α and VEGF expression, it is a candidate for treating wet AMD and other CNV-associated conditions.

## 1. Introduction

Age-related macular degeneration (AMD) is a leading cause of irreversible vision loss among people over the age of 50 in developed countries [1]. The advanced stage of wet AMD is represented by choroidal neovascularization (CNV), which is the abnormal ingrowth of new vessels under the macula. CNV develops following pathologic angiogenesis, including proteolysis of the extracellular matrix, endothelial cell migration and proliferation, and synthesis of new matrix components [2]. CNV can also occur along with other conditions including angioid streaks, pathologic myopia, and various inflammatory diseases [3]. Early treatments for CNV, such as laser photocoagulation, photodynamic therapy, and macular translocation, have yielded poor visual outcomes [4,5,6]. The recent development of pharmacological inhibitors of angiogenesis has focused on inhibiting vascular endothelial growth factor (VEGF) and potentially proteolytic enzymes such as matrix metalloproteinases [7,8,9]. New therapies that utilize VEGF antibodies, such as bevacizumab, ranibizumab, and aflibercept, have brought a paradigm shift to AMD treatment. However, these treatments have limitations [10,11,12] including the need for monthly clinic visits and frequent retreatments. Moreover, current intravitreal injection therapy with anti-VEGF agents is very expensive and time consuming. Thus, new treatments with fewer disadvantages are needed.

Flavonoids are natural compounds found in many fruits and vegetables that are often used as dietary supplements or herbal remedies. Interestingly, some flavonoids have been shown to inhibit endothelial cell migration and proliferation and tube capillary formation in vitro [13]. In addition, flavonoids are very safe and have low toxicity, making them potentially useful as chemo-prophylactic agents.

Chrysin (5,7-dihydroxyflavone), a flavonoid that is naturally present in many fruits and vegetables, has recently been shown to have anti-inflammatory, anti-oxidative, and anti-cancer effects; therefore, it has become an attractive therapeutic candidate in many medical research fields [14,15,16]. Although the exact mechanisms underlying the biological activities of chrysin remain unclear, recent evidence suggests that chrysin inhibits the transcriptional activation of VEGF, which is regulated in human tumor tissue by hypoxia-inducible factor-1 alpha (HIF-1α). Furthermore, chrysin has been shown to inhibit angiogenesis in nude mice [17] and it has no intraocular toxicity to rat retinas or rabbit lenticular tissue in or ex vivo, respectively [18,19].

In a preliminary proof-of-concept study, we tested the effects of chrysin in a small number of rats with experimentally induced CNV and showed that it significantly inhibited angiographic leakage [18]. Based on this initial result, we hypothesized that intravitreal injected chrysin may inhibit angiogenesis in an experimentally induced CNV. The present investigation was designed to further assess the inhibitory effects of intravitreally injected chrysin on angiogenesis. In addition, we measured HIF-1α and VEGF expression levels to elucidate the underlying mechanisms of chrysin-induced inhibition of angiogenesis.

## 2. Results

### 2.1. Effect of Chrysin on Mean Grade of Experimentally Induced CNV

We induced experimental CNV in the fundus of Brow Norway rats (*n* = 20) with a diode laser, with six or seven lesions concentrically placed around the optic disc in both eyes. One week after the laser treatment, a single intravitreal injection of chrysin solution (5 µL, 60 mM in 0.5% DMSO and balanced salt solution) was administered into the right eye, and the same volume of vehicle solution was administered into the left eye of each rat as a control. Two weeks after the laser treatment, fluorescein angiography was performed to evaluate CNV activity. The total numbers of analyzable CNV lesions were 124 and 128 in the chrysin-treated and control groups, respectively (*n* = 20 eyes/group). Each photocoagulated lesion was classified according to the amount of fluorescein leakage as follows: grade 1, minimum leakage or staining of tissue with no leakage; grade 2, small but evident leakage; grade 3, moderate intensity and medium-sized (diameter < 1/2 disc) leakage; or grade 4, large and evident leakage. Table 1 shows the number of CNV lesions with each angiographic grade in both groups. The mean CNV grade per lesion was significantly lower in the chrysin-treated group than in the control group (chrysin-treated: *n* = 124, variance = 1.30; control: *n* = 128, variance = 1.10; *p* < 0.001; Table 1).

### 2.2. Analysis of High-Leakage and Low-Leakage Groups of Experimentally Induced CNV Lesions

To further analyze the relationship between chrysin treatment and the degree of angiographic leakage, each photocoagulated lesion was categorized into a low- (grades 1 and 2) or high-leakage (grades 3 and 4) group. One week after intravitreal treatment with chrysin, significantly more CNV lesions in chrysin-treated eyes than those in control eyes were categorized into the low-leakage group (grades 1 and 2; *p* < 0.001). The relative risk (control eyes/chrysin-treated eyes) of the control group developing high-leakage lesions (grades 3 and 4) was 2.03 (95% confidence interval: 1.46–2.83) compared with the chrysin-treated group (Table 2).

### 2.3. Effect of Intravitreal Chrysin Administration on Growth and HIF-1α and VEGF Expression of Experimentally Induced CNV

**Figure 1:**
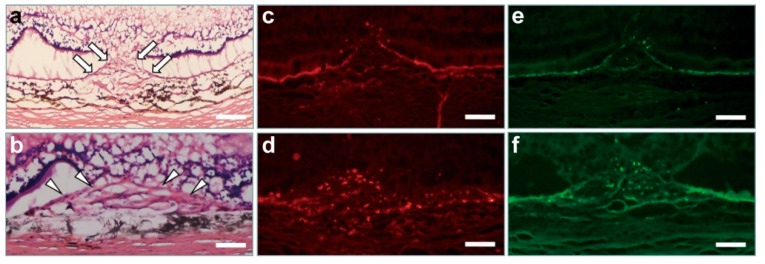
Hematoxylin and eosin and immunofluorescence staining of laser-induced CNV lesions. Typical samples of hematoxylin and eosin-stained sections show that chrysin-treated eyes (**a**) had much smaller CNV lesions (arrows) than did control eyes (arrowheads) (**b**). Immunofluorescence staining using mouse monoclonal hypoxia-inducible factor-1 alpha and rabbit polyclonal anti-rat vascular endothelial growth factor antibodies revealed minimal fluorescence in chrysin-treated eyes (**c,e**) compared with the enhanced fluorescence in control eyes (**d,f**). Scale bar = 50 μm. CNV, choroidal neovascularization.

**Figure 2:**
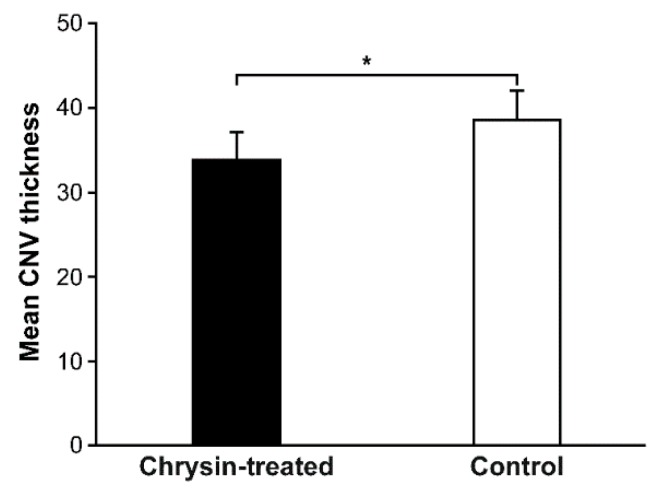
The thickness of experimental CNV lesions in chrysin-treated and control groups. Nine days after intravitreal chrysin treatment, the mean CNV thickness was compared between the chrysin-treated and control groups. CNV lesions in the chrysin-treated group were significantly thinner than those in the control group (*n* = 30 CNV lesions from 5 eyes/group; * *p* < 0.01, unpaired Student’s *t*-test). CNV, choroidal neovascularization.

Nine days after intravitreal treatment, retinal sections were analyzed using hematoxylin and eosin (H&E) and immunofluorescence staining. The mean CNV thickness in H&E-stained sections was significantly smaller in the chrysin-treated group than in the control group (*n* = 30 from 5 eyes/group; 33.90 ± 4.89 vs. 38.50 ± 5.43 μm, *p* < 0.001; Figure 1a,b and Figure 2). Immunofluorescence staining revealed reductions in HIF-1α and VEGF fluorescence in the retina/choroid complex of chrysin-treated eyes compared with control eyes (Figure 1c–f).

### 2.4. Intravitreal Administration of Chrysin Suppressed Expression of HIF-1α and VEGF in the Retina/Choroid Complex

**Figure 3:**
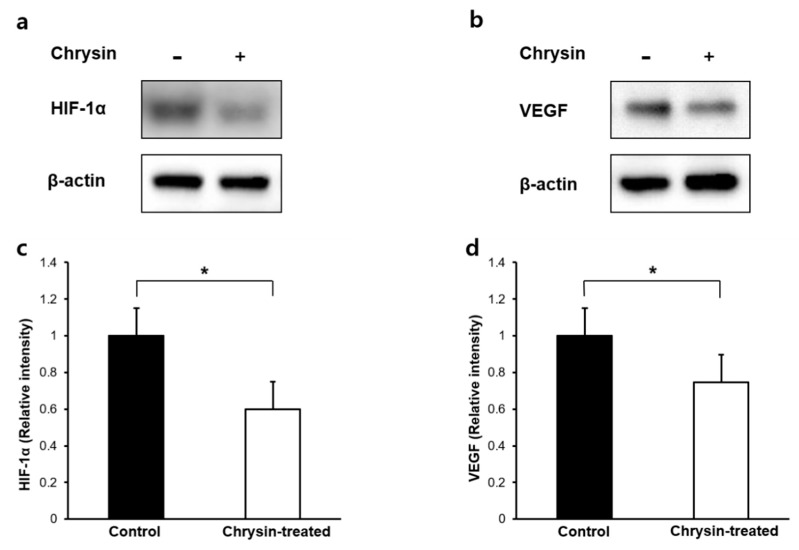
Effect of chrysin on hypoxia inducible factor (HIF)-1α and vascular endothelial growth factor (VEGF) expression in the chrysin-treated and control groups. HIF-1α (**a**) and VEGF (**b**) protein levels detected by immunoblotting showed that chrysin inhibited HIF-1α and VEGF protein expression. Relative band intensity of HIF-1α (**c**) and VEGF (**d**) protein normalized to β-actin revealed significantly decreased expression in the chrysin-treated group than in the control group (*n* = 15 eyes/group; * *p* < 0.001, Mann-Whitney U tests). Error bars indicate the standard error. HIF-1α, hypoxia inducible factor-1 alpha; VEGF, vascular endothelial growth factor.

**Figure 4:**
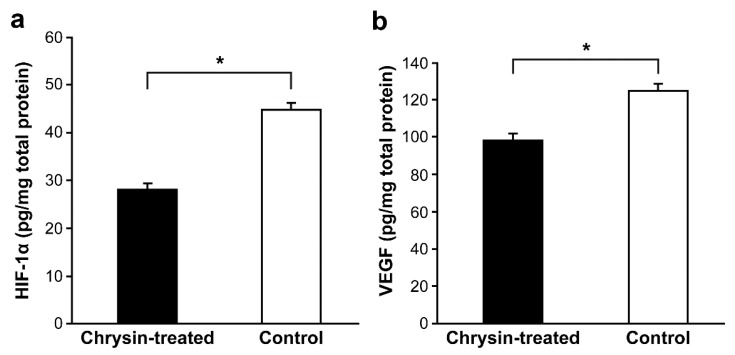
Concentrations of hypoxia inducible factor (HIF)-1α and vascular endothelial growth factor (VEGF) in the retina/choroid complex of the chrysin-treated and control groups. HIF-1α (**a**) and VEGF (**b**) protein levels were measured using enzyme-linked immunosorbent assays and normalized to the total protein concentration. The concentrations of both proteins were significantly lower in the chrysin-treated group than in the control group (*n* = 15 eyes/group; * *p* < 0.001, Mann-Whitney U tests). Error bars indicate the standard error. HIF-1α, hypoxia inducible factor-1 alpha; VEGF, vascular endothelial growth factor.

We next analyzed the expression of HIF-1α and VEGF proteins in the retina/choroid complex dissected from the remaining eyecup. Ten days after intravitreal treatment, chorioretinal concentrations of HIF-1α and VEGF were measured in 15 rats by Western blotting and enzyme-linked immunosorbent assay. Western blot results revealed reduced expression of HIF-1α and VEGF proteins in chrysin-treated eyes compared with control eyes (Figure 3a–d). Further enzyme-linked immunosorbent assay analysis showed a significantly lower mean HIF-1α concentration in the chrysin-treated group than in the control group (*n* = 15 eyes/group; 29.13 ± 1.19 vs. 46.40 ± 1.15 pg·mg^−1^ total protein, *p* < 0.001; Figure 4a). Similarly, there was a significant reduction in the mean VEGF concentration in the retina/choroid complex in the chrysin-treated group compared with that in the control group (*n* = 15 eyes/group; 97.93 ± 3.47 vs. 124.80 ± 5.13 pg·mg^−1^ total protein, *p* < 0.001; Figure 4b).

## 3. Discussion

The aim of this study was to determine the efficacy of a single intravitreal injection of chrysin on angiogenesis in an experimental rat model of CNV. The expression of HIF-1α and VEGF was also investigated to assess whether the chrysin-induced inhibition of angiogenesis was associated with a reduction in HIF-1α and VEGF expression in laser-induced CNV lesions. Our results demonstrated that eyes treated with chrysin exhibited substantially less leakage, as measured by fluorescein angiography than did eyes treated with vehicle solution. As the intravitreal injection of chrysin was shown to downregulate HIF-1α and VEGF expression in laser-induced CNV, it is postulated that the increased numbers of CNV lesions with grades 1 and 2 low leakage in the chrysin-treated group might result from a reduced expression of VEGF in laser-induced CNV. This causes a decreased level of new vessel growth and vascular leakage. In addition, eyes in the chrysin-treated group had notably reduced CNV thicknesses and less immunofluorescence in the neovascular tissue. These findings suggest that chrysin inhibits CNV development.

We injected chrysin solution in one eye and a vehicle in the control eye. It is a well-established method in animal study to inject the research drug into one eye and use the other eye as control. However, there is a possibility that chrysin injected in the right eye may have been absorbed into the systemic circulation, affecting the control eye. However, any drug injected intravitreally has a very low concentration in the blood. For example, following intravitreal injection, ranibizumab egresses into the systemic circulation and reaches its maximum serum concentration at approximately 12 h after administration. Its systemic-to-vitreous ratio was estimated to be 1:90,000 [20]. Therefore, the effective concentration of chrysin that may have affected the control eye would have been very low compared to that in the chrysin-treated eye.

CNV is the typical neovascular form of AMD. However, various diseases of the retina and choroid can also be accompanied by CNV as a secondary manifestation. The underlying pathogenesis of CNV has not been fully elucidated. Nevertheless, VEGF, a diffusible cytokine that promotes angiogenesis and vascular permeability, has been shown to play a key role. Evidence suggests that CNV development is promoted by increased VEGF expression [21,22]. The current standard care for CNV is treatment with anti-VEGF agents, which can maintain vision in most patients. Unfortunately, this approach has several drawbacks, including the need for frequent clinic visits and cost.

It was previously reported that VEGF expression is increased in both patients with AMD and animal models of laser-induced CNV [23]. In addition, *VEGF* gene transcription is mainly regulated by HIF-1, which is a heterodimeric transcription factor composed of 2α and 2β subunits. To activate *VEGF* gene transcription, HIF-1 binds to the hypoxia response element of the *VEGF* promoter region [24]. Furthermore, HIF-1α-mediated VEGF expression can be induced by growth factors, oncogenes, and hypoxia [25]. HIF-1 overexpression has been found in various human cancer tissues, and its level of activity correlates with angiogenesis and tumorigenicity [26]. Consequently, anti-angiogenic therapies for CNV have targeted the HIF-1α/VEGF system.

Dietary intake of antioxidants and other supplements has been demonstrated to slow the progression of advanced AMD [27,28]. Plant-based dietary interventions, including with flavonoids, can modify the cancer risk and prevent other chronic diseases [14,29]. Studies have also shown that flavonoids regulate cell proliferation and in vitro angiogenesis in tumor tissues, in addition to endothelial cell function in laser-induced CNV [30,31].

Chrysin is a flavonoid that is naturally present in many fruits and vegetables and has been studied as a potential antitumor agent [32]. In a previous pilot study, we demonstrated that chrysin inhibits angiographic leakage in laser-induced CNV [18]. The present investigation further examined the inhibitory effects of an intravitreal injection of chrysin on angiogenesis and HIF-1α and VEGF expression within laser-induced CNV lesions. The chrysin-treated group showed significantly lower HIF-1α and VEGF expression in the retina/choroid complex than did the control group. Our results are consistent with those of previous in vitro studies of tumor tissues showing that chrysin inhibits *VEGF* gene transcription by inhibiting HIF-1α expression [17]. Several prior studies have also reported that chrysin downregulates the expression of protein kinase B and nuclear factor-κB [33,34,35]. Further studies are warranted to investigate the effects of chrysin on the expression of downstream genes in an animal model of CNV.

Chrysin can affect HIF-1α synthesis and degradation in different ways. The phosphatidylinositol 3-kinase/protein kinase B signaling pathway plays an important role in HIF-1α expression [36]. Interestingly, chrysin inhibits AKT phosphorylation, which in turn suppresses HIF-1α expression. Chrysin also reduces the half-life and stability of HIF-1α. Prolyl hydroxylation of HIF-1α at the oxygen-dependent degradation domain is critical for maintaining HIF-1α at the steady-state level [37]. Chrysin promotes prolyl hydroxylation of the HIF-1α oxygen-dependent degradation domain, thus facilitating its ubiquitination and subsequent proteasomal degradation [17]. Finally, HIF-1α is stabilized by binding to heat shock protein 90. Chrysin inhibits this binding, which interferes with the interaction between HIF-1α and heat shock protein 90, ultimately promoting HIF-1α degradation [17]. Therefore, chrysin reduces HIF-1α levels both by inhibiting its production and by promoting its degradation. The net effect is suppression of the VEGF expression that is needed for CNV development.

This study has several limitations. First, we injected a relatively high concentration of chrysin (15 mg·mL^−1^, corresponding to ~60 mM). We used this concentration because chrysin is known to have poor bioavailability. This concentration is higher than the predicted minimum dose necessary to have an effect; similar small-molecule inhibitors impede CNV at concentrations between 10 μM and 1 mM. Therefore, the effects of different chrysin concentrations on CNV require further investigation. Second, we used 0.5% dimethyl sulfoxide (DMSO) in the buffer solution because of the poor solubility of chrysin. This concentration of DMSO may have had a suppressive effect on CNV. However, the same concentration and amount of DMSO solution was used as the vehicle solution and injected into the control eyes. Furthermore, statistical analyses revealed considerable reductions in the mean CNV grade, the relative risk of developing high-leakage lesions, and concentrations of HIF-1α and VEGF, as well as thinner CNV membranes, in chrysin-treated eyes compared to control eyes. Therefore, it is unlikely that these changes were due to DMSO suppression. Third, intravitreal administration of chrysin was performed one week after laser photocoagulation in this study, which was not consistent with the protocol that indicated that intravitreal injection was to be performed during anesthesia just after laser induction [38]. The substance studied was injected at various time points after laser photocoagulation, from day 0 to day 16, since previous studies showed that CNV first appeared on day 7 after laser treatment, reaching its peak on day 21 in rodent model of CNV. Recent research using optical coherence tomography angiography (OCTA) confirmed that CNV first appeared on day 7 after photocoagulation with a progressive number of new vessels with increased size and structure until day 21 [39]. Therefore, we injected chrysin one week after placing the laser to accurately check the inhibition effect of chrysin on the development of new vessels. Finally, it is possible that bias was introduced during the CNV grading process. Determining the CNV grade via fluorescein angiography images was sometimes difficult even though the presence of a CNV lesion could be easily identified. We minimized this bias by using two examiners who were blind to the rat group and treatment to determine the CNV grades and by using the higher grade if there was a difference in grading between examiners. The activity of experimentally induced CNV is routinely evaluated with its leakage on fluorescein angiography. Although fluorescein angiography has been the most useful tool for imaging the presence of CNV in rodents, sometimes the image quality may be not good enough to fully evaluate CNV because of optic problems, cataract formation, and prominent leakage. In these situations, indocyanine green angiography could have supplemented fluorescein angiography. Recently, a few studies implementing OCTA for imaging CNV in animal models have been published [39,40]. Shah et al. [40] used visible light-OCTA to study laser-induced CNV at different time-points after laser injury to monitor CNV development and measure CNV lesion size. They reported that compared to isolectin-stained choroidal flatmounts, visible light-OCTA is a more reliable tool for both detecting CNV development and accurately measuring the size of CNV lesions in a mouse model of laser-induced CNV. Additionally, in their study comparing the utility of OCTA and conventional fluorescein angiography for the quantitative analysis of the retinal and choroidal vasculature in a rat model of laser-induced CNV, Meyer et al. [39] showed that OCTA enables a more detailed evaluation of CNV development. Given its superior resolution and non-invasiveness relative to conventional fluorescein angiography, OCTA can be used to analyze the response of CNV to new therapeutic agents in further in vivo studies.

In summary, our findings show that chrysin inhibits laser-induced experimental CNV in rats. To the best of our knowledge, this is the first study to yield in vivo data demonstrating that chrysin suppresses HIF-1α and VEGF expression in CNV lesions. Collectively, our results support the view that chrysin has the potential to be used as a therapeutic agent for neovascular AMD and other CNV-associated conditions. Preventing CNV development is becoming increasingly important because the number of patients with neovascular AMD is increasing due to the aging of populations in Western countries. AMD is therefore likely to become a more serious healthcare burden in the future. Current strategies for preventing AMD, such as the use of dietary supplements, have been shown to reduce the risk of progression; however, additional preventive strategies are needed [27,28]. Our results suggest that chrysin, a common and readily available natural flavonoid, may be a candidate substance for the treatment and prevention of CNV in AMD and other CNV-associated diseases. Future investigations should focus on evaluating the potential of chrysin as a treatment for neovascular AMD and other CNV-associated vision-threatening conditions.

## 4. Materials and Methods

### 4.1. Materials

Chrysin (purity > 96%) and DMSO (purity ≥ 99.9%) were purchased from Sigma-Aldrich (St. Louis, MO, USA). Sodium fluorescein was purchased from Alcon Laboratories, Inc. (Fort Worth, TX, USA). Primary and secondary antibodies used for immunofluorescence microscopy were purchased from Santa Cruz Biotechnology Inc. (Santa Cruz, CA, USA) and Invitrogen (Carlsbad, CA, USA), respectively.

### 4.2. Laser-Induced Experimental Model of CNV

The study protocol was approved by the Ajou University Ethics Committee for Animal Experiments (protocol number: 2018–0026). Male Brown Norway rats (*n* = 20), 7–9 weeks of age and weighing 200–250 g, were used in this study in accordance with the Association for Research in Vision and Ophthalmology Statement on the Use of Animals in Ophthalmic and Vision Research. All procedures complied with Animal Research: Reporting of In Vivo Experiments (ARRIVE) guidelines and were performed in accordance with the National Institutes of Health guide for the care and use of laboratory animals (NIH Publications No. 8023, revised 1978). Rats were anesthetized via an intramuscular injection of a 1:1 mixture of tiletamine/zolazepam (10 mg·kg^−1^) and xylazine (5 mg·kg^−1^) for all procedures. The techniques and laser parameters used to establish the experimental CNV model were adapted from our previous study, with slight modifications [18]. In brief, pupils were dilated with 1% tropicamide and 2.5% phenylephrine. The fundus was visualized using slit-lamp biomicroscopy with a slide cover glass and 2.5% hydroxypropyl methylcellulose solution (Methocel; Ciba Vision, Wessling, Germany). Using a frequency-doubled, diode-pumped, solid-state laser (Visulas 532s; Carl Zeiss Meditec Inc., Dublin, CA, USA) with a wavelength of 532 nm, spot size of 100 µm, 100 ms exposure, and 150 mW power, 6 or 7 lesions were concentrically placed approximately equidistant around the optic discs in both eyes of each rat. The rupture of Bruch’s membrane was defined by the presence of acute vapor bubbles. Lesions with subretinal hemorrhage that interfered with evaluation or adjacent merged lesions were excluded from analyses.

### 4.3. Intravitreal Chrysin Administration

One week after laser treatment, rats were anesthetized, and a single intravitreal injection of chrysin solution (5 µL, 60 mM in 0.5% DMSO and balanced salt solution) was administered into the right eye using a 30-gauge needle. The same volume of vehicle solution was administered into the left eye of each rat as a control.

### 4.4. Fluorescein Angiography

**Figure 5:**
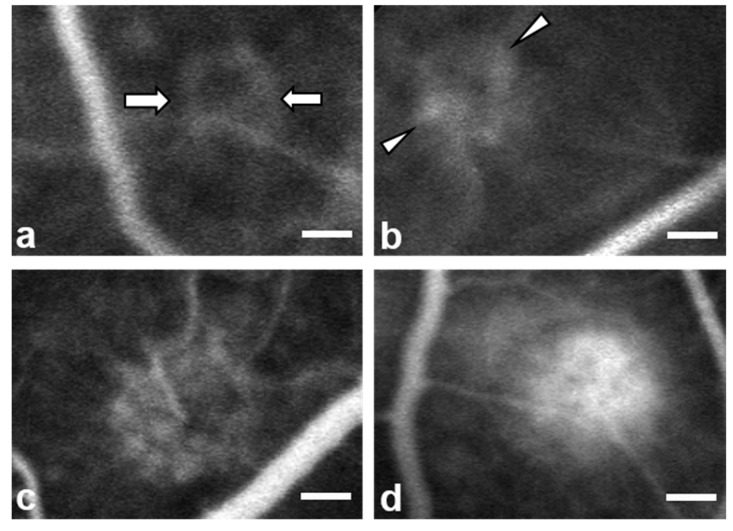
Fluorescein angiography images showing standard examples of each choroidal neovascularization grade after laser treatment in the rat retina. Each photocoagulated lesion was graded (1–4) according to the size and intensity of fluorescein dye leakage. (**a**) Grade 1, minimum leakage or staining of tissue with no leakage (arrows). (**b**) Grade 2, small but evident leakage (arrowheads). (**c**) Grade 3, moderate intensity and medium-sized (diameter < 1/2 disc) leakage. (**d**) Grade 4, large and evident leakage. Scale bar = 50 μm.

Two weeks after laser treatment, fluorescein angiography was performed using a confocal scanning laser ophthalmoscope (Heidelberg Retinal Angiograph 2; Heidelberg Engineering, Heidelberg, Germany) to evaluate CNV development and activity. The angiography procedure was adapted from our previous study [18]. Each rat was injected with 0.3 mL of 10% fluorescein sodium through the tail vein, and then early phase (<2 min) and late phase (>7 min) angiography was performed. CNV formation was evaluated according to the size and intensity of dye leakage using a scoring system modified from a previous study [41]. Lesions were considered “leaky” if hyperfluorescence was observed during the early phase of angiography and if the size and intensity of the leakage increased during the late phase. The images that were acquired ~7 min after fluorescein sodium injection were consistently selected for determining the grade of CNV leakage. Each photocoagulated lesion was classified according to the amount of fluorescein leakage as follows: grade 1, minimum leakage or staining of tissue with no leakage; grade 2, small but evident leakage; grade 3, moderate intensity and medium-sized (diameter < 1/2 disc) leakage; or grade 4, large and evident leakage (Figure 5). Lesions were graded by two examiners (J.H.S. and K.Y.M.), who were blind to the animal number and experimental group. If the two examiners did not agree on the grade for a particular lesion, the higher grade was used in the analysis. The number of CNV lesions with each angiographic grade in both groups and the mean CNV grade per lesion was evaluated. To further analyze the relationship between chrysin treatment and the degree of angiographic leakage, each photocoagulated lesion was categorized into a low- (grades 1 and 2) or high-leakage (grades 3 and 4) group. The numbers of low- or high-leakage lesions in each group and the relative risk of the control group developing high-leakage lesions were analyzed.

### 4.5. Histological Analysis and Immunofluorescence

Sixteen days after laser treatment and 9 days after chrysin injection, 5 rats were euthanized, and histological analyses were conducted. Whole-animal perfusion fixation was performed with 4% paraformaldehyde. Following this, the eyes were enucleated and fixed in 4% paraformaldehyde for 6 h. Next, the anterior segment was removed, and eyecup samples were dehydrated and embedded in paraffin. Serial sagittal sections (5 µm thick), which included all lesions, were prepared and stained with H&E. The sections were then assessed under a light microscope (Carl Zeiss, Jena, Germany) to determine the effects of chrysin on CNV growth. The maximal CNV thickness was measured from the outer border of the pigmented choroidal layer to the highest point of the CNV membrane in the carefully selected central section of each CNV lesion. Sections were also analyzed via immunofluorescence using mouse primary monoclonal HIF-1α and rabbit primary polyclonal anti-rat VEGF antibodies, respectively (Santa Cruz Biotechnology Inc. Santa Cruz, CA, USA). Secondary immunofluorescent antibodies were Alexa 555 and Alexa 488 for HIF-1α and VEGF, respectively (Invitrogen, Carlsbad, CA, USA).

### 4.6. Quantification of HIF-1α and VEGF in the Retina and Choroid

Seventeen days after laser treatment and 10 days after chrysin injection, chorioretinal concentrations of HIF-1α and VEGF were measured in 15 rats. The animals were euthanized, and enucleation was performed. The globes were kept on ice until dissection, and the dissected pieces were frozen in liquid nitrogen and stored at −80 °C before further immunochemical analysis. The dissection was performed by removing the anterior segment and cutting the frozen globe in half through the optic nerve. The remaining eyecup samples were further dissected under a microscope into the vitreous, retina/choroid, and sclera. The retina/choroid complex was sonicated indirectly in lysis buffer (Sigma-Aldrich, St. Louis, MO, USA) for 15 min. The total protein concentration was calculated using the Bradford protein assay. Equal amounts of the samples (50 μg) were resolved with sodium dodecyl sulfate polyacrylamide gel electrophoresis, and then transferred to nitrocellulose membranes. HIF-1α and VEGF protein were detected by immunoblotting using the specific antibodies mentioned above and β-actin was used as a loading control. Band signals were detected by chemiluminescence (ImageQuant LAS 4000 mini, GE Healthcare, Chicago, IL, USA). The Western blotting bands were quantified by ImageJ (National Institute of Health, Bethesda, MD, USA). HIF-1α and VEGF protein concentrations in the supernatant were measured using an enzyme-linked immunosorbent assay kit (R&D Systems, Minneapolis, MN, USA) and normalized to the total protein concentration.

### 4.7. Statistical Analysis

Based on the results of our previous study [18], we estimated that the sample size required to determine a difference of 15%–20% in the mean CNV grade between groups, using unpaired Student’s *t*-tests at a significance level of *p* < 0.05 and a power of 90%, should be 119 CNV lesions per group. The sample size was increased to 130 CNV lesions per group to account for non-assessable lesions. To obtain this number, 20 rats with 6 or 7 lesions induced in each eye were used. As described in the methods above, the right and left eye of each rat was treated with chrysin and vehicle solution, respectively. Therefore, no randomization or allocation process was necessary.

The mean CNV grades and thicknesses are expressed as the mean ± standard deviation. Pearson’s chi-squared tests were performed to compare differences between the low- and high-leakage groups of CNV grades. HIF-1α and VEGF concentrations are expressed as the mean ± standard error. Unpaired Student’s *t*-tests and Mann–Whitney U tests were used to compare chrysin- and vehicle-treated eyes for the CNV grade/thickness and HIF-1α and VEGF concentrations, respectively. Data sets are available as Supplementary Materials (Spreadsheets S1) submitted along with the manuscript. All analyses were performed using SPSS version 23.0 (IBM Corp., Armonk, NY, USA). Differences were considered significant at *p* < 0.05.

## 5. Conclusions

The number of patients with neovascular AMD is rapidly increasing and AMD is becoming a more serious healthcare burden. The present findings show that chrysin inhibited laser-induced CNV and downregulated HIF-1α and VEGF expression. These findings suggest that chrysin is a candidate substance to be used as a therapeutic or preventive agent for neovascular AMD and other CNV-associated diseases. Future studies are warranted to evaluate the potential of chrysin as a treatment for CNV-associated vision-threatening conditions.

## Figures and Tables

**Table 1:** Number of CNV lesions in each grade and mean CNV grade of chrysin-treated and control rat eyes.

	CNV Grade					Mean CNV Grade
	1	2	3	4	Total	
Chrysin-treated Eyes	37	30	29	28	124	2.39 ± 1.14
Control Eyes	19	15	45	49	128	2.97 ± 1.05
*p* value ^1^						<0.001

The mean CNV grade data ± standard deviation is reported; ^1^ Unpaired Student’s *t*-test; CNV, choroidal neovascularization.

**Table 2:** Number of CNV lesions in the low-leakage and high-leakage groups.

	Low-Leakage Group	High-Leakage Group	Total
Chrysin-treated Eyes	67	57	124
Control Eyes	34	94	128
*p* value ^1^			<0.001

^1^ Pearson’s chi-squared test; CNV, choroidal neovascularization.

## Supplementary Materials

Supplementary materials can be found at https://www.mdpi.com/1422-0067/21/8/2842/s1.

## Abbreviations

AMD	Age-related Macular Degeneration
CNV	Choroidal Neovascularization
DMSO	Dimethyl Sulfoxide
H&E	Hematoxylin and Eosin
HIF-1α	Hypoxia-inducible Factor-1α
OCTA	Optical Coherence Tomography Angiography
VEGF	Vascular Endothelial Growth Factor
