# Supplementary material for: Inhibition of Hypoxia-Inducible Factor-1α and Vascular Endothelial Growth Factor by Chrysin in a Rat Model of Choroidal Neovascularization
Source: Int J Mol Sci. 2020 Apr 18;21(8):2842. doi: 10.3390/ijms21082842 (PMC7215732; doi:10.3390/ijms21082842)
Supplement: Supplementary file 1 [file ijms-21-02842-s001.pdf]

Table 1. HIF-1 α Relative Band Intensity.

HIF-1α Relative Band Intentisy		
HIF	Control	Chysin-treated
	1.1	0.45
	1.3	0.4
	0.65	0.65
	0.9	0.5
	1	0.6
	1.05	0.75
	0.85	0.55
	0.95	0.8
	1.2	0.75
	1.35	0.6
	0.7	0.7
	1.15	0.7
	0.8	0.45
	1.25	0.5
	0.75	0.6
Mean HIF Relative Intensity	1	0.6

Table 2. VEGF Relative Band Intensity.

VEGF Relative Band Intensity		
VEGF	Control	Chysin-treated
	0.95	0.65
	1.3	0.7
	1.05	0.75
	0.85	0.55
	1.1	0.85
	1.15	0.8
	1.2	0.7
	0.9	0.55
	1.15	0.95
	0.7	0.75
	0.85	0.95
	1	0.75
	0.8	0.6
	1.05	0.75
	0.95	0.9
Mean VEGF Relative Intensity	1	0.75

Table 3. The Mean HIF Level.

The Mean HIF Level		
HIF	Chrysin-treated	Control
	24	45
	32	52
	28	39
	26	48
	22	46
	27	50
	26	49
	36	39
	33	49
	29	42
	39	53
	32	47
	25	46
	28	50
	30	41
Mean HIF level	29.13333	46.4

Table 4. The Mean VEGF Level.

The Mean VEGF Level		
VEGF	Chrysin-treated	Control
	121	138
	115	142
	90	163
	96	141
	84	109
	101	134
	79	121
	88	105
	95	136
	112	94
	97	107
	108	98
	106	116
	103	123
	74	145
Mean VEGF level	97.93333	124.8
